# Cytokine release syndrome after bronchoalveolar lavage

**DOI:** 10.1186/s12890-023-02704-0

**Published:** 2023-10-16

**Authors:** Margaret Guerriero, Feras Ally, Keith R. Loeb, Viswam S. Nair

**Affiliations:** 1 Division of Pulmonary, Critical Care & Sleep Medicine, University of Washington School of Medicine, Seattle, WA USA; 2 Department of Laboratory Medicine and Pathology, University of Washington School of Medicine, Seattle, WA USA; 3 Division of Public Health Sciences, Fred Hutchinson Cancer Center, Seattle, WA USA; 4 Clinical Research Division, Fred Hutchinson Cancer Center, 1100 Fairview Ave N, Seattle, WA 98109 USA

**Keywords:** Bronchoalveolar lavage, Cytokine release syndrome, Bone marrow transplant, Organizing Pneumonia

## Abstract

**Background:**

Immunosuppressed bone marrow transplant patients with pulmonary infiltrates routinely undergo bronchoscopy with bronchoalveolar lavage (BAL) to investigate potential etiologies. Cytokine release syndrome after BAL is unreported in the literature in general and in this patient population.

**Case presentation:**

We report on an allogeneic bone marrow transplant patient with non-infectious organizing pneumonia of the lungs who developed delayed and rapidly progressive shock and hypoxia post-procedure over the course of 12 h resulting in intensive care unit admission for supportive care. BAL was characterized by a marked lymphocytic, cytotoxic T cell infiltrate on pathology and flow cytometry without clear evidence of infection. The patient’s clinical status improved quickly only after the initiation of high dose intravenous steroids and returned to baseline as an outpatient.

**Conclusion:**

The patient’s clinical data and course suggest a cytotoxic T cell response from the lung and BAL as the etiology. With an increasing number of cellular therapies for cancer entering the clinic, the potential for unusual but morbid complications from routine bronchoscopy should be considered.

## Background

The aim of this case report is to present the clinical course and associated data from bronchoalveolar lavage (BAL) in a patient with organizing pneumonia from an allogeneic stem cell transplant who decompensated after routine bronchoscopy. We identify a likely etiology for the patient’s rapid and profound deterioration that is unreported in the literature.

## Case presentation

**Fig. 1:**
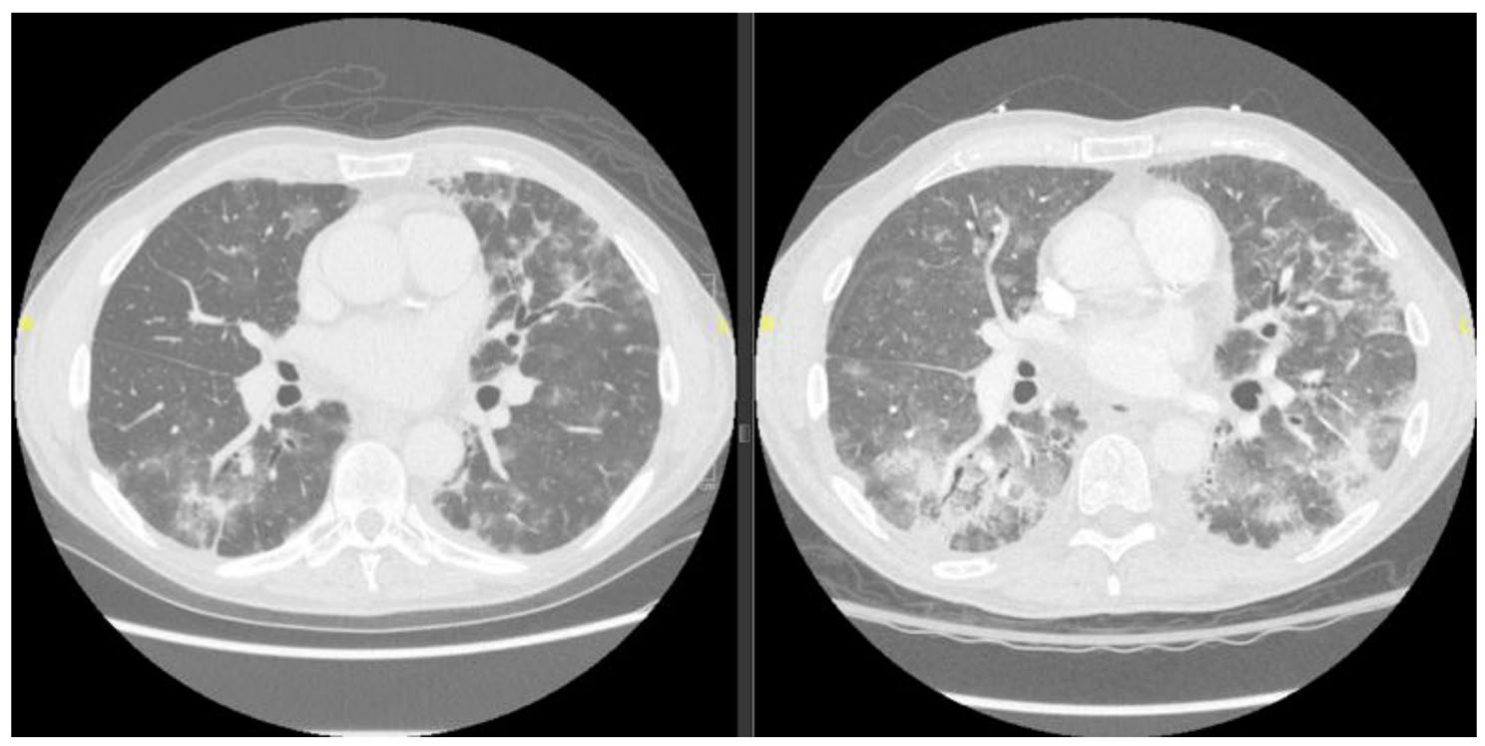
Chest CT scan at clinic (left) and at hospital admission 11 days later (right)

A 69-year-old male 9 months removed from an allogeneic mismatched unrelated bone marrow transplant for myelodysplastic syndrome presented to our pulmonary clinic for evaluation of new pulmonary infiltrates (Fig. 1). He had been doing well from a transplant standpoint, and sirolimus immunosuppression was discontinued two months prior. Three months prior, the patient presented to the ED, tested positive for COVID, and was discharged home with an unremarkable course. His home antigen test cleared and his repeat viral swab two months later showed no evidence of persistent COVID.

The patient was a non-smoker without a pulmonary history, and other than a cough was not in distress and saturating well on room air. A bronchoscopy was ordered for further investigation. When the patient arrived for the procedure 11 days later, he had an increased cough. His saturations prior to the procedure were normal but dropped during moderate sedation induction with fentanyl and versed on 4 L per minute (lpm) of oxygen. His airways appeared grossly normal by white light bronchoscopy. A BAL was obtained from the inferior segment of the lingula, with 37 of 120 ml lavagate returned. The fluid was white, turbid, and non-purulent. Post procedure, the patient did well and was weaned off oxygen in 30 min. At that point he developed coughing, hypoxia, and a drop in blood pressure.

**Fig. 2:**
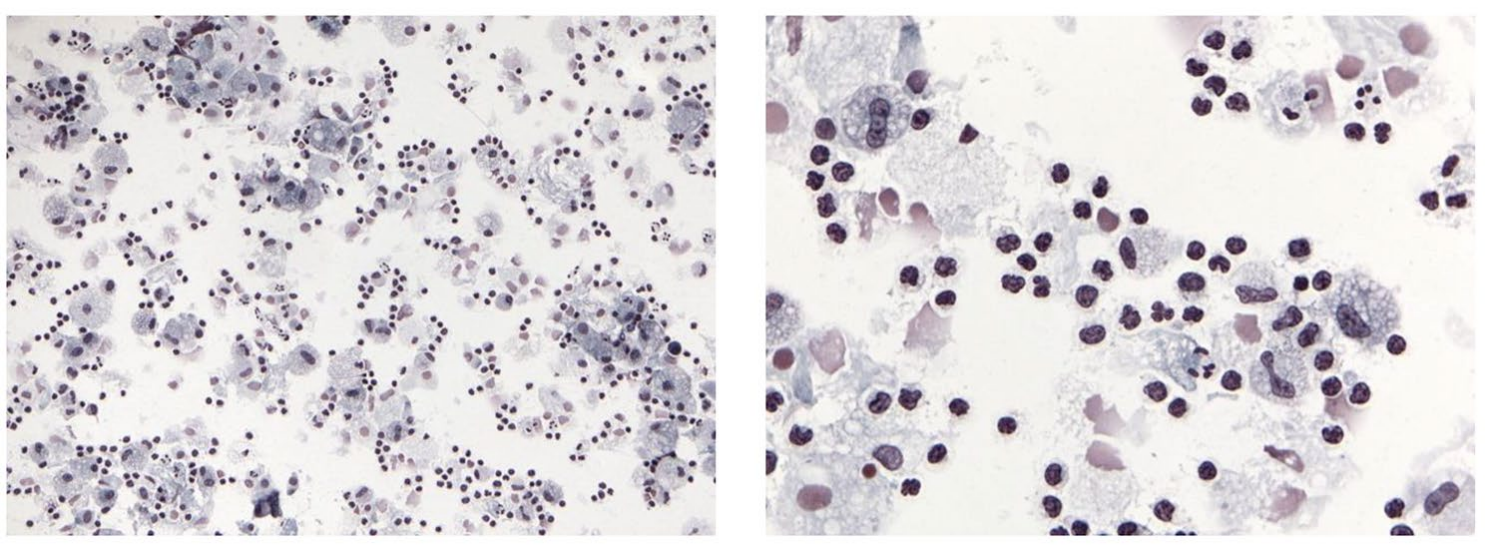
BAL microscopy showing reactive T cells at low (left) and high (right) power

**Fig. 3:**
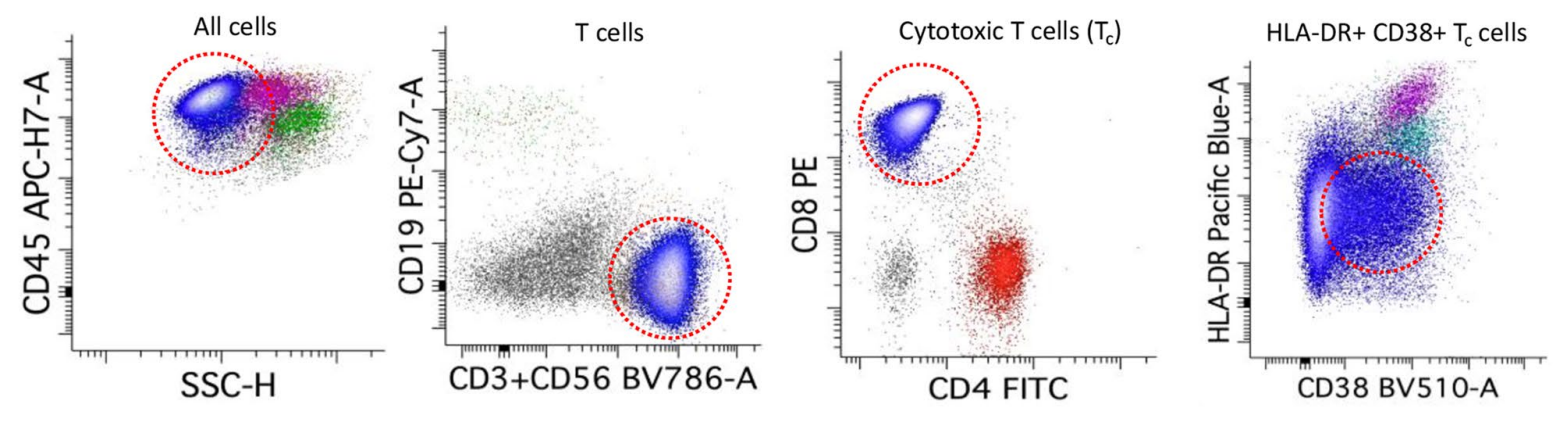
BAL flow cytometry of mature, cytotoxic T cells expressing CD38 and HLA-DR

He was transferred to the acute care evaluation floor of the cancer center, where his temperature rose to 38.4^0^ C and his blood pressure dropped to 80/50 mmHg while on 4–6 lpm of oxygen. He was conversational but mildly altered. Blood cultures were drawn, fluids administered, and antibiotics initiated. On arrival to the emergency department from the cancer center, the patient continued to require oxygen from 8 to 10 lpm, with mean arterial pressures < 65 mmHg after 3 L of intravenous fluids (IVF). Bloodwork was notable for a leukocytosis with neutrophilia and a bedside echocardiogram was normal. A repeat CT showed progression of infiltrates compared to 2 weeks prior (Fig. 1). The patient was admitted to the ICU for vasopressor support and observation. Over the next 24 h BAL and blood culture data were sterile and the patient remained on low dose vasopressors and high dose oxygen requirements. The BAL was notable for a cell differential of 23% Macrophages, 4% Neutrophils, 72% Lymphocytes, and 1% Eosinophils. Pathology of BAL showed numerous lymphocytes with reactive features (Fig. 2) that were primarily cytotoxic T-cells with an increased CD8:CD4 ratio of 7:1 and variable expression of activation markers CD38 and HLA-DR (Fig. 3). A BAL COVID PCR antigen test was negative. Following a multi-disciplinary discussion of the most likely diagnosis, methylprednisone 2 mg/kg was initiated. Over the course of the next 48 h the patient improved remarkably with vasopressors and oxygen weaned to off. All cultures from BAL and blood remained negative. He was discharged on 1 mg/kg of prednisone daily and recovered within the ensuing weeks to baseline without disease recrudescence over the next 6 months during prednisone tapering.

## Discussion & conclusions

Non-infectious or “cryptogenic” organizing pneumonia in bone marrow transplant patients who receive allogeneic donor cells is considered to be a form of graft dysfunction–although not formally reported as graft vs. host disease of the lung–that is mediated by T cells [1]. Cytokine release syndrome (CRS) post bronchoscopy is unreported in this situation.

While post-bronchoscopy pyrexia is relatively common and mediated by TNFα and interleukin [2, 3], the severity of this patient’s presentation warrants reporting. We posit that a lack of systemic immunosuppression, and potentially a recent history of COVID, led to a robust T cell response after bronchoscopy. Supportive data for this hypothesis include a pronounced 72% lymphocyte population on the BAL differential (normally 5–10%) and a highly skewed CD8:CD4 ratio [normally 1:1.5, [4]] with reactive T cells, all in the setting of a negative microbiologic workup. Importantly, HLA-DR + CD38 + cytotoxic T cells are upregulated in viral illnesses like influenza and COVID [5, 6], along with hyperinflammatory disorders [7], to further support this hypothesis. Furthermore, a delayed onset of decompensation after the patient had fully recovered from conscious sedation is consistent with our clinical experience of CRS. It is probable that the BAL itself caused either (A) a local inflammatory response (B) a systemic delivery of cells or (C) a combination of these two things to cause CRS. In addition, this patient received topical lidocaine to the airways during the procedure, and derivatives of lidocaine delivered by this method may potentiate the inflammatory cascade [2].

Management of CRS draws from the evolving use of chimeric antigen receptor T (CAR-T) cells as salvage therapy for lymphoma and includes supportive care with IVF in a ward or ICU setting, systemic steroids intravenously, and the use of biologic agents [8]. We considered the latter on day two of our patient’s hospital course but his excellent response to steroids eliminated the need for treatment escalation. Other etiologies that are possible since this diagnosis was strictly clinical include aspiration leading to culture negative sepsis, idiosyncratic sirolimus induced lung toxicity [9], or idiopathic pneumonia syndrome. However, the most likely diagnosis remains a CRS like decompensation post bronchoscopy based on the data and patient’s course. Since the use of cellular immunotherapies will increase and expand from academic centers into the community over time, we felt it important to report this novel and highly morbid complication after BAL to raise awareness.

## Data Availability

Data sharing is not applicable to this article as no datasets were generated or analysed during the current study.
